# Case Report: Button battery ingestion—an underestimated emergency in children

**DOI:** 10.3389/fped.2024.1484458

**Published:** 2025-01-22

**Authors:** Karin Konzett, Stefanie Gang, Lukas Poyntner, Eberhard Reithmeier, Susanne Dertinger, Burkhard Simma

**Affiliations:** ^1^ Department of Pediatrics, Academic Teaching Hospital, Landeskrankenhaus Feldkirch, Feldkirch, Austria; ^2^ Department of Otorhinolaryngology, Academic Teaching Hospital, Landeskrankenhaus Feldkirch, Feldkirch, Austria; ^3^ Department of Anaesthesiology and Critical Care Medicine, Academic Teaching Hospital, Landeskrankenhaus Feldkirch, Feldkirch, Austria; ^4^ Department of Pathology Academic Teaching Hospital, Landeskrankenhaus Feldkirch, Feldkirch, Austria

**Keywords:** button batteries, fatal outcome, aortoesophageal fistula, chocking hazard, button cell ingestion

## Abstract

In general, the battery-related emergency department visit rate has continued to rise in the last decade. We present two cases of previously healthy toddlers (14 and 24 months old) with unwitnessed battery ingestion, who presented with massive, hematocrit-relevant hematemesis. Initially, both children showed stable vital signs. Following a symptom-free interval, both had a recurrence of massive hematemesis, which could not be controlled despite a multidisciplinary approach with pediatric, radiology, ENT specialists, endoscopy and anesthesia. Pathological workup showed necrosis with secondary aortoesophageal fistula due to battery-induced colliquation necrosis caused by caustic soda produced at the minus pole. We conclude, that preclinical risk scores, excellent clinical pathways (e.g., from Children's Hospital of Philadelphia) and detailed approaches from the National Capital Poison Center in the USA and also the European Society of Pediatric Gastroenterology Hepatology and Nutrition (ESPGHAN) offer clear and concise instructions for the management of button battery ingestion, but clinical awareness for vascular complications must be heightened. A multidisciplinary treatment algorithm for this fatal complication should be implemented and trained in pertinent hospitals. Moreover, it is of great importance to raise awareness for button battery ingestion in educational campaigns for parents and caregivers.

## Introduction

Foreign body ingestion (FBI) was the fourth leading cause for calls to poison control centers in the United States in 2019 (1). The battery-related emergency department (ED) visit rate per 100,000 children has continued to rise in the last decade and was two times higher between 2010 and 2019 than between 1990 and 2009 (2). The batteries were most frequently intended for watches, laptops, toys and games, followed by hearing aids, remote controls or flashlights (2). Button batteries (BB) of larger diameter (>20 mm diameter) and high voltage (3-volt lithium) ingested by children <4 years were considered to be the most concerning cause of life-threatening complications (3, 4). Complicated courses are mainly vocal cord paralysis, but also esophageal perforation and tracheoesophageal or aortoesophageal fistulas (4). When battery ingestion is witnessed, medical consultation and therapy are time-critical issues. Because of the broad variation of clinical presentation, also unwitnessed cases of battery ingestion should be kept in mind.

We now present two cases of BB ingestion with fatal consequences. Viewpoints from the current literature are incorporated in the Discussion.

## Case presentations

The first patient was a healthy 2-year-old girl, who presented after two episodes of hematemesis that morning, with epistaxis primarily thought to be the cause. Emergency transport service was called. At arrival in the ED vital signs were: temperature 35.3°C, heart rate 153/min, blood pressure 87/50 mmHg (P5 70/22 mmHg), capillary refill time was 3 s, SpO_2_ 100%, GCS 12. After volume therapy (25 mL/kg, balanced electrolyte solution), the patient's condition improved. *Bleeding had stopped spontaneously and hemoglobin (HB) controls showed a dilution effect (initial HB 9.3 g/dL to 7.5 g/dL 1.5 h later)*. After two hours of inconspicuous monitoring on the pediatric ward massive hematemesis unexpectedly recurred. The patient was treated with crystalloid fluids (25 mL/kg), red blood cell transfusion (25 mL/kg, push-and-pull method) and tranexamic acid (30 mL/kg). To identify the bleeding source, the patient was transferred to the operating room (OR). During transport, her condition again deteriorated. After problem-free intubation under continuous infusion of blood components the ENT specialist was able to rule out a nasopharyngeal bleeding source. Blood seemed to spill up from the esophagus. The esophagus was packed and the endoscopy team was called. Before their arrival, cardiac arrest occurred and cardiopulmonary resuscitation (CPR) had to be started. Under ongoing CPR and continuous administration of blood components and clotting agents the endoscopic team was unable to bring the situation under control. With sustained asystolia and wide and unreactive pupils the team decided to stop resuscitation efforts after 35 min of CPR.

**Figure 1:**
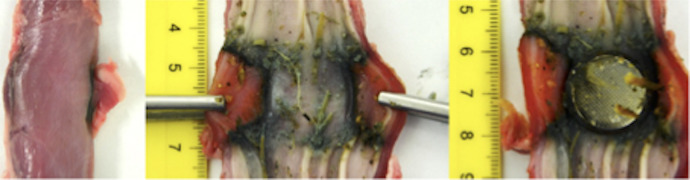
Excerpt from the experimental series of our pathology department: Porcine esophagus, colliquative necrosis in the esophagus due to a CR2032 BB determined as the cause.

At autopsy, a 20-mm token-shaped necrotic lesion was found in the esophagus, *which opened into an aortoesophageal fistula. The histological workup showed a colliquative necrosis, but the battery could not be found*. After a literature research and concordant experiments on porcine esophagi, colliquative necrosis of the esophagus by production of caustic sodium induced at the minus pole (anode) of a CR 2032 BB was defined as the initiating cause (Figure 1).

**Figure 2:**
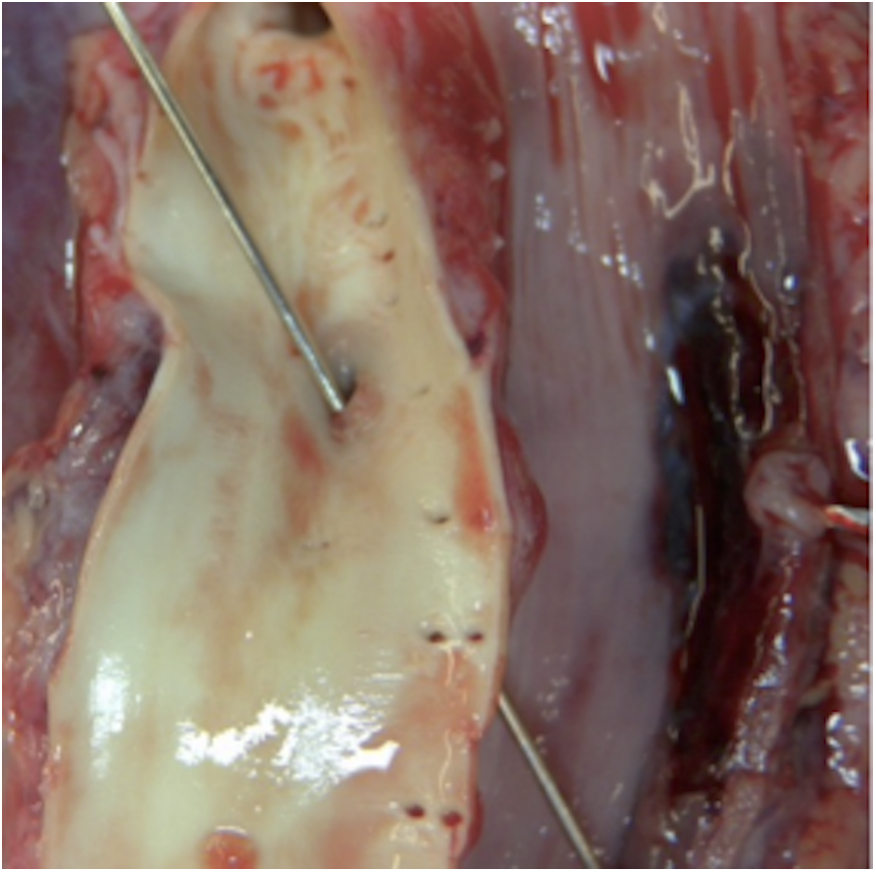
Case 2: Aortoesophageal fitsula (of our 14 month old patient), which was deemed the cause of recurrent fulminant bleeding.

The second patient was a healthy 14-month-old boy, who presented with *torrential* hematemesis and respiratory failure in an affiliated hospital. He was intubated and transferred to our pediatric intensive care unit. Before transfer, we recommended an x-ray because of the many similarities to patient 1. A BB (halo sign, diameter >22 mm) was detected in the lower gastrointestinal tract. On arrival in our resuscitation bay the patient was cardiorespiratory stable [vital signs: heart rate 140/min, blood pressure 107/57 mmHg (P5 71/21 mmHg), capillary refill time 3 s, SpO_2_ 98%]. A central and an arterial line were installed, and balanced fluids were administered with prompt and sustained improvement. Gastroscopy showed a coin-shaped ulceration in the esophagus 14 cm aboral, with no signs of acute bleeding; morphologically it corresponded to necrosis caused by a BB. The CT scan showed minimal air leak outside the upper third of the esophagus, indicating a perforation. The mediastinum showed signs of inflammation. Although angiography showed no abnormalities, transport to an extracorporeal membrane oxygenation (ECMO) and cardiothoracic surgery center was organized. At the very moment of discharge, massive oral bleeding recurred and the patient went into cardiac arrest. CPR was administered, massive transfusion was initiated, and a catheter was placed in the esophagus and blocked until the endoscopy team arrived. Return of spontaneous circulation was achieved in less than 5 min. Given the dynamics of the situation, the two-hour ground-based transport was cancelled and the boy was transferred to the OR. Extended thoracotomy was performed, esophagus and aorta were inspected carefully. Other than a minor arterial esophageal branch, no active bleeding site or accompanying hematoma was found. Unsure whether the bleeding problem was already solved, the blocked catheter was deflated and about ten minutes later massive bleeding recurred. Immediate reblocking, further massive transfusion, and ongoing coagulation and catecholamine management led to intermittent but only partial resuscitation and recurrence of spontaneous circulation. After 45 min of CPR, the patient was declared dead because of hemorrhagic shock. The autopsy showed an aortoesophageal fistula, which was deemed the cause of the recurrent fulminant bleeding (Figure 2).

## Discussion

Fatal hemorrhage after BB ingestion is a rare event, but has been described with increasing frequency (2, 5–8). In both our cases the battery ingestion was unwitnessed. Most major events were related to unwitnessed ingestion (56%) (4). Classic symptoms, such as difficulty swallowing, drooling, fever, cough or nausea (9) were neither presented by these patients nor reported by their parents. Rather atypically, the leading symptom in our patients was hemodynamically relevant nose bleeding and hematemesis. Retrospectively, these bleeding events must be interpreted as so-called “sentinel bleeding” (5, 10) usually caused by aortoesophageal fistulas. Even that assumption suggests that the condition is life-threatening, requiring a prompt and multidisciplinary approach (11, 12). Case reports (7) show, that symptom free intervals can last several weeks. In a recent overview study of 13 cases Lanzafame et al. (7) demonstrate that rapid diagnosis and management are crucial for the infants' survival. According to their literature review, only ten cases of sentinel bleeding have been managed successfully with subsequent patient survival.

Fortunately, vascular injuries caused by BB ingestion remain a rarity and long-lasting symptom-free intervals are not uncommon. In an overview by Akinkugbe et al. of 361 cases of severe complications following BB impaction, only 51 (14%) were vascular in nature. However, 61% of children who died after BB impaction had vascular injuries and 75% of vascular injuries were aortoesophageal fistulas (13).

Battery size (>20 mm), high voltage (>3 V), prolonged time of impaction (>2–3 h) and young age (<4–6 years) (13, 14) are known as predictive factors for poor outcome. When a BB is lodged in the esophagus, severe mucosal damage can occur within 2–4 h and therefore immediate endoscopic removal is imperative. Of children under the age of 6 who ingested batteries >20 mm size 14% had a severe or fatal outcome (4). Various mechanisms play a role in the injury process. Pressure necrosis *per se* is caused by the presence of the battery in the narrowing distal esophagus. Electrical discharges cause localized colliquation necrosis by producing caustic sodium and hydrogen gas at the minus pole (anode) (15, 16).

To reduce any damage, the oral application of honey has recently been proposed in the literature (11, 14, 17). It is recommended that 10 mL of honey (2 teaspoons) be administered every 10 min (17) up to six times (11) between ingestion and removal of the battery in children older than 1 year. In fact, clinical trials and experimental studies are very limited (18). A recent literature review by Schmidt et al. (18) revealed only four studies on the administration of honey after BB ingestion. Three of these presented experimental *in vitro* and *in vivo* results, and one reported a clinical retrospective study of eight patients, of whom only two received honey before button battery removal (19). Honey may be protective due to its neutralizing effect on the battery-induced pH change and the formation of a shielding film around the BB thanks to honey's greater viscosity. But also other agents like sucralfate, or even saline show benefits in reducing local damage (18). It is argued that the resulting lower pH leads to less damage (13). Although in our ED we see many children with FBI and also with ingested batteries, the cases described above were the first of their kind. According to the literature BB ingestions are on the rise, with rising numbers of fatal outcomes.

Excellent flow chart diagrams, e.g., from CHOP (17), the National Capital Poison Center in the USA (14) and the ESPGHAN position paper in Europe (11) offer clear and concise instructions for the management of witnessed or even suspected BB ingestions, but clinical awareness for aortoesophageal fistulas must be enhanced. A button battery impaction score has also been introduced as a clinical tool for the potential referral of children to an appropriate medical facility (12). In our opinion, a multidisciplinary treatment algorithm (pediatric, radiology, ENT specialists, endoscopy, anesthesia) (8, 11, 20) for this potentially fatal complication should be implemented and trained in pertinent hospitals. Moreover, it is of the utmost importance to raise awareness for BB ingestion in educational campaigns for parents and caregivers.

## Conclusion

When unclear *torrential* nasal bleeding or hematemesis occurs, battery-related damage must be given consideration and a time-critical, multidisciplinary approach is imperative. It is crucial that the bleeding source be investigated *with a CT angiography or an endoscopy*, even if the patient is cardiorespiratory stable. In the case of a sentinel hemorrhage with suspected aortoesophageal fistula, a symptom-free interval is not unusual and should be used to plan further steps for interventional or surgical care. Both treatment methods require established pathways, either in-house (equipment, pediatric interventionalist, pediatric cardiac surgeon) or with transport to an appropriate center.

## Data Availability

The original contributions presented in the study are included in the article/Supplementary Material, further inquiries can be directed to the corresponding author.
