# Supplementary material for: Case Report: Button battery ingestion—an underestimated emergency in children
Source: Front Pediatr. 2025 Jan 22;12:1484458. doi: 10.3389/fped.2024.1484458 (PMC11793998; doi:10.3389/fped.2024.1484458)
Supplement: Supplementary file 1 [file Table1.docx]

**Supplement 1**: Histological Workup of the Aortoesophageal Fistula, Case 1


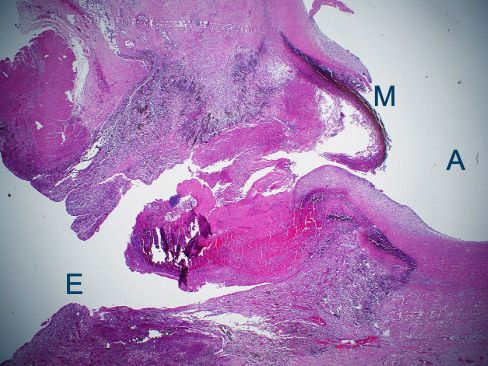


Abbreviations: E… esophagus, A… aorta, M… tunica muscularis aortae
