# Supplementary figures and images for: Case Report: Button battery ingestion—an underestimated emergency in children
Source: Front Pediatr. 2025 Jan 22;12:1484458. doi: 10.3389/fped.2024.1484458 (PMC11793998; doi:10.3389/fped.2024.1484458)

**Supplement 2:** Timeline Case Reports

Case 1


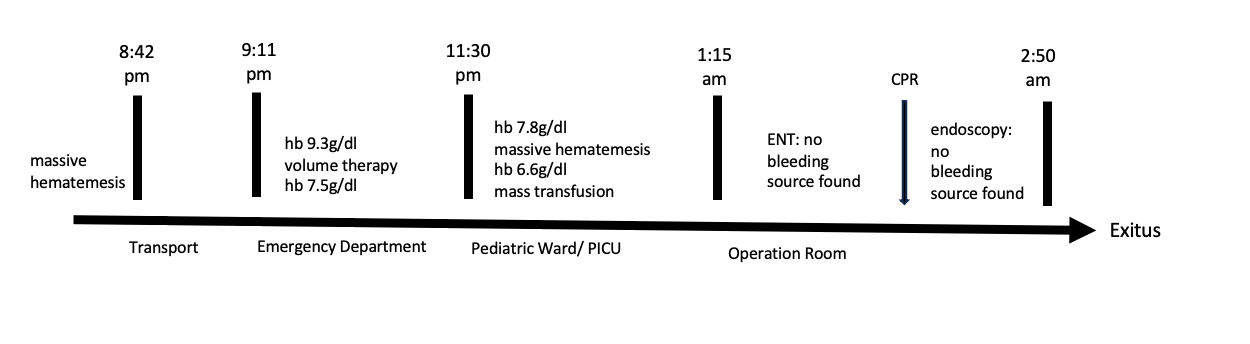


Case 2
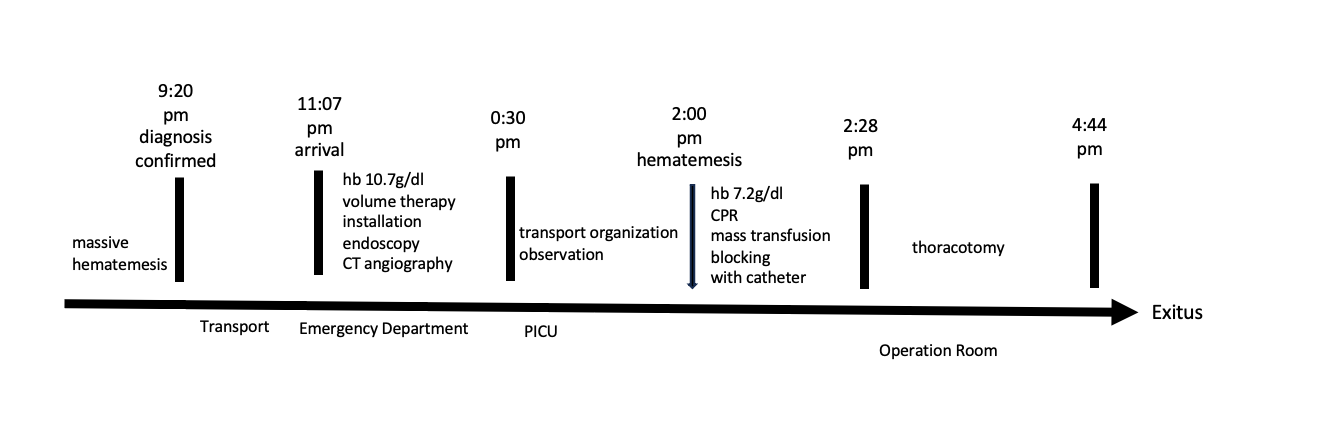

Supplement: Supplementary file 2 [file Table2.docx]
